# Smoking status before and after colorectal cancer diagnosis and mortality in Korean men: A population‐based cohort study

**DOI:** 10.1002/cam4.3609

**Published:** 2020-11-24

**Authors:** Doeun Jang, Sunho Choe, Ji Won Park, Seung‐Yong Jeong, Aesun Shin

**Affiliations:** ^1^ Department of Preventive Medicine Seoul National University College of Medicine Seoul Korea; ^2^ Cancer Research Institute, Seoul National University Seoul Korea; ^3^ Division of Colorectal Surgery, Department of Surgery Seoul National University College of Medicine Seoul Korea

**Keywords:** colorectal neoplasms, mortality, prognosis, tobacco smoking

## Abstract

**Background:**

Smoking is a well‐known risk factor for colorectal cancer incidence; however, the effect of smoking before and after cancer diagnosis on mortality has not been addressed well. Thus, we aimed to evaluate the association of prediagnosis and postdiagnosis smoking status and mortality among colorectal cancer patients.

**Methods:**

A retrospective cohort consisted of 37,079 male colorectal cancer patients. Smoking status was defined from information within 2 years of colorectal cancer diagnosis for prediagnosis and at least 1 year later for postdiagnosis. The prediagnostic and postdiagnostic smoking status were categorized into four groups (nonsmoker/nonsmoker, nonsmoker/smoker, smoker/nonsmoker, and smoker/smoker). Hazard ratios (HRs) and 95% confidence intervals (CIs) were estimated using the Cox proportional hazard model.

**Results:**

During a median of 6.3 years of follow‐up, a total of 3980 deaths and 2137 deaths from colorectal cancer occurred. The number of prediagnosis smokers were 11,100 and 62.4% of them quitted smoking after the diagnosis. Significantly elevated mortality rate in prediagnosis smokers was observed regardless of postdiagnosis smoking status (smoker/nonsmoker [HR, 1.30; 95% CI, 1.20–1.41] and smoker/smoker [HR, 1.21; 95% CI, 1.09–1.34]). Among patients treated with surgical operation only, those who quit smoking after diagnosis showed lower mortality rates compared to continual smokers (HR, 0.80; 95% CI, 0.67–0.96).

**Conclusions:**

Smoking before cancer diagnosis rather than postdiagnosis has stronger impact on prognosis colorectal cancer patients, and quitting smoking may improve survival, especially among early stage colorectal cancer patients.

## Graphical abstract

This study found that both smoking before and after cancer diagnosis is associated with increased mortality rate among colorectal cancer patient. Quitting smoking after diagnosis was significantly related to diminished mortality among colorectal cancer patients receiving only operation, who is considered as early stage colorectal cancer patients, though smoking cessation after diagnosis among colorectal cancer patients who received radiotherapy or chemotherapy was not associated with significantly decreased mortality.

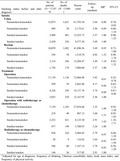

## 1 INTRODUCTION

According to GLOBOCAN 2018, colorectal cancer is the third most common cancer and fourth leading cause of cancer death among men worldwide.^1^ In Korea, colorectal cancer is the third most commonly diagnosed cancer (16,672 newly diagnosed colorectal cancer cases in 2016) and the fourth leading cause of cancer death (4659 colorectal cancer deaths in 2016) among men.^2^

Smoking is a well‐established risk factor for colorectal cancer incidence,^3^, ^4^, ^5^ and smoking was the leading risk factor for men and was responsible for a large proportion of the cancer burden in men in 2017.^6^ Smoking leads to increased mortality as well as to an increased risk of colorectal cancer incidence. Smoking leads to poor prognosis when current smokers are compared with nonsmokers.^7^ People who are continual smokers after cancer diagnosis are at higher risk for future cancers and death.^7^ In the 2014 U.S. Surgeon General’s Report, tobacco smoking was said to lead to premature death, and these results apply to cancer patients as well as survivors.^7^ In a previous study, elevated colorectal cancer‐specific mortality for current smokers compared with nonsmokers was observed among men, and colorectal cancer‐specific mortality risk in former smokers decreased significantly with duration since cessation.^8^ Smoking after diagnosis among colorectal cancer patients was associated with a significant increase in the risk of death compared to not smoking after diagnosis.^9^

As cancer therapy has improved and patients with colorectal cancer have increased, it becomes important to estimate the association between lifestyle behavior and mortality among patients diagnosed with colorectal cancer. After colorectal cancer diagnosis, patients may be recommended to make lifestyle changes to improve prognosis. Previous studies reported that 24%–44% of patients who smoked at cancer diagnosis succeeded in quitting smoking.^10^, ^11^, ^12^ In Schnoll study,^10^ smoking cessation was measured after 3 months from baseline. In Burke study,^11^ time of smoking cessation was not clear, although information on former smoking was collected at prediagnosis and postdiagnosis. The Mayer study^12^ was case‐control design and cases were answered to the question about postdiagnosis former smoking. However, it is unclear whether changes in smoking behavior affect mortality. There are some studies that assess the association between smoking status before colorectal cancer diagnosis and mortality,^13^ but few studies investigate the changes in smoking status from prediagnosis to postdiagnosis. Therefore, we conducted this study to investigate the association of smoking status before and after colorectal cancer diagnosis and mortality among Korean men with colorectal cancer.

## 2 MATERIALS AND METHODS

### 2.1 Study population

**FIGURE 1:**
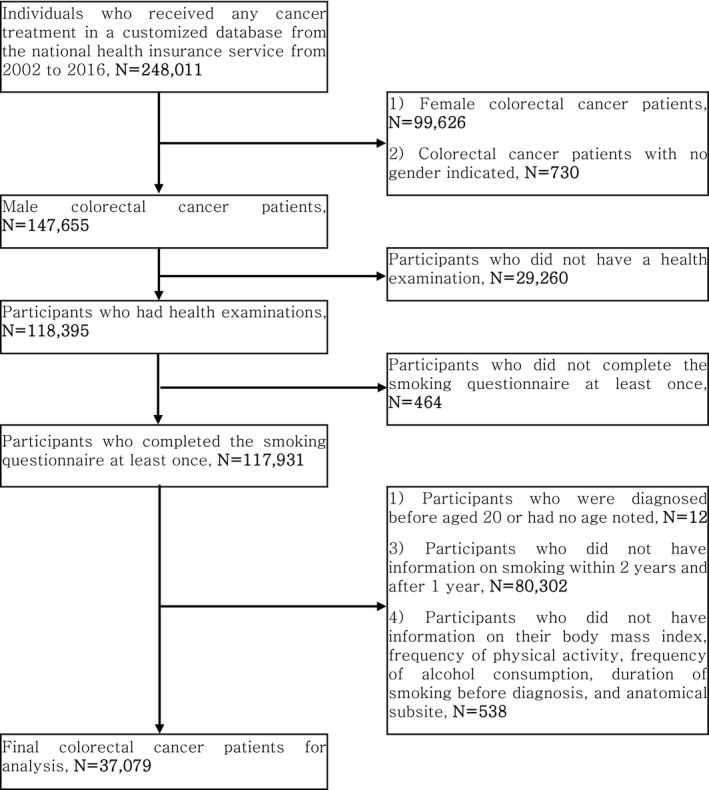
Selection of the study population

This study was conducted based on the National Health Insurance Services (NHIS) customized database, which is a retrospective cohort based on claim data.^14^ The NHIS is a mandatory single insure in Korea which covers approximately 97% of the Korean population (3% were covered by Medicare) and contains medical resource utilization information, demographic characteristics, and health examinations from 2002 to 2017. The colorectal cancer cases were defined based on the 10th International Classification of Diseases (ICD‐10) diagnostic code corresponding to colorectal cancer (C18–20) and the claim code of its treatment (Table S1).^15^ We selected subjects diagnosed with colorectal cancer who had undergone health examinations. We excluded colorectal cancer patients who did not complete the smoking questionnaire at least once and who were diagnosed before age 20, had missing age, did not have information on smoking at least within 2 years and after 1 year of diagnosis, had missing information on body mass index (BMI), physical activity, alcohol use, duration of smoking, and subsite. Finally, 37,079 participants were included in the final analysis (Figure 1). This study protocol was approved by the Institutional Review Board (IRB) at Seoul National University Hospital (IRB number 1906‐116‐1041).

### 2.2 Data collection

Data on smoking status, duration of smoking (years), frequency of alcohol intake, and frequency of physical activity were collected by self‐reported questionnaires during health examinations biannually. Anthropometric indices, including height and weight, were measured in a health‐care center. BMI was calculated as the subject’s weight (kg) divided by the square of the subject’s height (m^2^). BMI was categorized into four groups (<18.5; 18.5–24.9; 25.0–29.9; and ≥30) based on the World Health Organization classification. As we used claim data, we had limited information to assess the tumor stage of the colorectal cancer patients. Therefore, we categorized the subjects into three groups by the treatment they had received, based on the idea that early stage patients are likely to be treated only with surgery, while advanced‐stage patients tend to be treated with chemotherapy or radiotherapy without surgery.^16^ In this study, we used the type of treatment to reflect the cancer stage of colorectal cancer patients (Figure S1). Smoking status (smoker vs. nonsmoker) was reported at prediagnosis and postdiagnosis. Participants were grouped according to the prediagnosis and postdiagnosis smoking status into four groups (nonsmoker/nonsmoker, nonsmoker/smoker, smoker/nonsmoker, and smoker/smoker). Information on the duration of smoking was collected at the prediagnosis health examination and categorized into four groups (<10, 10–19, 20–29, and ≥30 years). We used claim data within 1 year of colorectal cancer diagnosis when calculating the Charlson comorbidity index using Quan's coding algorithms.^17^ Death date and cause of death data among subjects were individually linked to each subject using Statistics Korea data. Colorectal cancer‐specific deaths were identified using ICD‐10 codes (C18–20). Participants were followed until death or 13 July 2017, whichever occurred first. In an analysis regarding the association between prediagnosis smoking status and mortality, the follow‐up duration was calculated from the date of diagnosis to death or the end of the follow‐up. When we performed analyses about the postdiagnosis smoking status or change of smoking status and mortality, the follow‐up duration was calculated from the date of postdiagnosis health examination to death or the end of the follow‐up.

### 2.3 Statistical analysis

The characteristics of participants were compared by conducting the chi‐squared test for categorical variables and *t*‐test or analysis of variance (ANOVA) for numerical variables. We used a Cox proportional hazards regression model to evaluate the association between smoking status and all‐cause and colorectal cancer‐specific mortality among colorectal cancer patients by calculating hazard ratios (HRs) and 95% confidence intervals (95% CIs).

Age at diagnosis with colorectal cancer, BMI, frequency of physical activity, frequency of alcohol intake, treatment, and Charlson comorbidity index were considered to be potential confounders; thus, they were included in the model for adjustment. Health examinations were used within 2 years before colorectal cancer diagnosis and 1 year after colorectal cancer diagnosis because we assumed that it would take time for smoking behavior to affect the participant mortality. To confirm the differences by treatment, analyses were stratified by treatment. Because a previous study reported that there was a different association between smoking and overall mortality by anatomic site,^18^ we conducted stratified analyses by subsite, that is, colon (C18) versus rectum (C19–20). In the sensitivity analyses, we restricted subjects who had health examinations within 1 year before colorectal cancer diagnosis to reflect smoking status shortly before colorectal cancer diagnosis. All statistical analyses were conducted using SAS software version 9.4 (SAS Institute Inc.), and figures were generated using STATA SE 14.

## 3 RESULTS

During the median follow‐up of 6.3 years (245,542 person‐years), 3980 all‐cause deaths and 2137 colorectal cancer specific‐deaths among colorectal cancer patients occurred. The median duration from prediagnosis smoking status assessment to colorectal cancer diagnosis was 0.37 years, and that from cancer diagnosis to postdiagnosis smoking status assessment was 2.53 years. Compared to the patients who were nonsmokers before colorectal cancer diagnosis, those who were smokers were younger and more likely to have low BMI and drink alcohol frequently (all *p*‐values <0.001). Over 65% of colorectal cancer patients underwent surgery only without chemotherapy or radiotherapy regardless of smoking status (Table 1). The proportions of participants who continued to smoke after diagnosis and who quit after diagnosis among colorectal cancer patients were 11.3% and 18.7%, respectively.

**TABLE 1:** Characteristics according to smoking status before colorectal cancer diagnosis

Characteristics before diagnosis	Nonsmoker		Smoker	
	(*N* = 25,979)		(*N* = 11,100)	
Age at diagnosis, years				
<50	2,319	(8.9)	1,912	(17.2)
50–59	6,321	(24.3)	3,748	(33.8)
60–69	9,563	(36.8)	3,680	(33.2)
≥70	7,776	(29.9)	1,760	(15.9)
BMI before diagnosis				
<18.5	456	(1.8)	376	(3.4)
18.5–24.9	15,318	(59.0)	7,279	(65.6)
25.0–29.9	9,511	(36.6)	3,209	(28.9)
≥30.0	694	(2.7)	236	(2.1)
Frequency of physical activity before diagnosis (per week)				
0	11,895	(45.8)	5,644	(50.9)
1–2	6,187	(23.8)	2,897	(26.1)
≥3	7,897	(30.4)	2,559	(23.1)
Frequency of alcohol intake before diagnosis (per week)				
<1	13,099	(50.4)	3,491	(31.5)
1–2	6,953	(26.8)	3,733	(33.6)
3–4	3,324	(12.8)	2,204	(19.9)
≥5	2,603	(10.0)	1,672	(15.1)
Treatment				
Operation	17,738	(68.3)	7,379	(66.5)
Operation with radiotherapy or chemotherapy	7,377	(28.4)	3,229	(29.1)
Radiotherapy and chemotherapy	864	(3.3)	492	(4.4)
Charlson comorbidity index				
0	1,453	(5.6)	790	(7.1)
1	3,228	(12.4)	1,512	(13.6)
2	3,527	(13.6)	1,610	(14.5)
≥3	17,771	(68.4)	7,188	(64.8)
Subsites				
Colon	15,564	(59.9)	6,228	(56.1)
Rectum	10,415	(40.1)	4,872	(43.9)

Abbreviation: BMI, body mass index.

Compared to not smoking before colorectal cancer diagnosis, smoking before colorectal cancer diagnosis was associated with significantly increased mortality (HR, 1.23; 95% CI, 1.14–1.32). Subjects who had smoked 10–19 years before diagnosis showed the highest mortality among smokers (1.48 [1.19–1.83]). Significantly elevated mortality was found in the population of patients who smoked after colorectal cancer diagnosis compared to the population who were not smokers after diagnosis (1.15 [1.05–1.26]) (Table 2).

**TABLE 2:** Association between prediagnosis and postdiagnosis smoking status and all‐cause mortality among male colorectal cancer patients

Smoking status	No. of patients (*N* = 37,079)	No. of deaths (*N* = 3,980)	Person‐years	Follow‐up (median)	HR^a^	95% CI
Prediagnosis						
Nonsmoker	25,979	2,819	173,026.02	6.36	1.00	
Smoker	11,100	1,161	72,516.93	6.20	1.23	1.14–1.32
Duration of smoking, years^b^						
<10	5,180	437	27,081.25	5.16	1.25	1.12–1.38
10–19	1,209	88	8,552.68	6.95	1.48	1.19–1.83
20–29	2,536	195	16,833.66	6.33	1.40	1.20–1.63
≥30	2,175	441	20,049.34	9.46	1.12	1.01–1.24
Postdiagnosis						
Nonsmoker	32,058	3,450	131,120.29	3.63	1.00	
Smoker	5,021	530	20,072.28	3.58	1.15	1.05–1.26
Prediagnosis/postdiagnosis						
Nonsmoker/nonsmoker	25,134	2,703	103,690.09	3.66	1.00	
Nonsmoker/smoker	845	116	3,686.39	3.99	1.30	1.08–1.56
Smoker/nonsmoker	6,924	747	27,430.20	3.54	1.30	1.20–1.41
Duration of smoking, years^b^						
<10	3,133	279	9,233.50	2.62	1.23	1.09–1.40
10–19	813	62	3,733.26	4.00	1.50	1.16–1.94
20–29	1,671	149	6,836.01	3.64	1.58	1.33–1.87
≥30	1,307	257	7,627.43	6.23	1.22	1.07–1.39
Smoker/smoker	4,176	414	16,385.89	3.55	1.21	1.09–1.34
Duration of smoking, years^b^						
<10	2,047	158	5,965.80	2.70	1.17	1.00–1.38
10–19	396	26	1,656.83	3.70	1.62	1.10–2.39
20–29	865	46	3,466.05	3.59	1.10	0.82–1.48
≥30	868	184	5,297.21	6.58	1.23	1.06–1.43

^a^ Adjusted for age at diagnosis, frequency of drinking, Charlson comorbidity index, body mass index, frequency of physical activity, and treatment.

^b^ Prediagnosis duration of smoking.

When prediagnosis/postdiagnosis smoking status was considered in the analysis, a significant increase in mortality among participants who started smoking after colorectal cancer diagnosis compared to participants who had never smoked was observed (1.30 [1.08–1.56]). Additionally, patients who quit smoking after colorectal cancer diagnosis, smokers/nonsmokers in terms of the prediagnosis/postdiagnosis smoking status, showed significantly elevated mortality compared with those who did not quit smoking (1.30 [1.20–1.41]) (Table 2). These patterns, the increased mortality among prediagnosis and postdiagnosis smokers, persisted in the sensitivity analyses including subjects who had smoking status assessment within 1 year before a colorectal cancer diagnosis (Table S2). Patients who were exposed to tobacco for 10–29 years before colorectal cancer diagnosis had higher mortality (10–19 years: 1.48 [1.19 to 1.83]; 20–29 years: 1.40 [1.20–1.63]). Furthermore, mortality was also elevated among patients who had continually smoked before and after colorectal cancer diagnosis (1.21 [1.09–1.34]). Among subjects who had smoked before diagnosis and continued smoking after diagnosis, patients who smoked 10–19 years showed the highest mortality, although the differences between groups were not marginally significant (1.62 [1.10–2.39]) (Table 2). There was no significant association between the prediagnosis or postdiagnosis smoking status and the colorectal cancer‐specific mortality. Regarding the change in smoking status, the smoker/nonsmoker group showed significantly elevated colorectal cancer‐specific mortality (1.21 [1.08–1.35]) (Table S3).

In the stratified analysis according to subsite, quitting smoking after diagnosis was associated with increased mortality among both colon cancer patients (1.06 [0.90–1.25]) and rectal cancer patients (1.10 [0.92–1.32]), although not statistically significant. For rectal cancer patients, starting smoking after colorectal cancer diagnosis was not significantly related to increased mortality (1.32 [0.98–1.78]). For the stratified analysis according to cancer treatment, both prediagnosis nonsmoking and postdiagnosis nonsmoking had a relation to decreased mortality among colorectal cancer patients who underwent surgery only (0.61 [0.52–0.70] for nonsmokers/nonsmokers; 0.86 [0.65–1.15] for nonsmokers/smokers; and 0.80 [0.67–0.96] for smokers/nonsmokers). However, associations between prediagnosis/postdiagnosis smoking status and mortality among colorectal cancer patients who underwent surgery with radiotherapy or chemotherapy were different from those among colorectal cancer patients who underwent surgery only. Briefly, quitting smoking after colorectal cancer diagnosis was significantly associated with increased mortality only among colorectal cancer patients who underwent surgery with radiotherapy or chemotherapy (1.42 [1.18–1.71]) (Table 3).

**TABLE 3:** Association between smoking status change and all‐cause mortality among male colorectal cancer patients by subsite and treatment

Smoking status before and after diagnosis	No. of patients (*N* = 37,079)	No. of deaths (*N* = 3,980)	Person‐years	Follow‐up (median)	HR^a^	95% CI
Subsite						
Colon						
Nonsmoker/nonsmoker	15,075	1,621	61,793.54	3.64	0.80	0.70–0.92
Nonsmoker/smoker	489	58	2,175.61	3.94	0.89	0.66–1.18
Smoker/nonsmoker	3,808	401	15,225.73	3.57	1.06	0.90–1.25
Smoker/smoker	2,420	236	9,377.30	3.49	1.00	
Rectum						
Nonsmoker/nonsmoker	10,059	1,082	41,896.56	3.69	0.85	0.72–1.00
Nonsmoker/smoker	356	58	1,510.78	4.02	1.32	0.98–1.78
Smoker/nonsmoker	3,116	346	12,204.47	3.49	1.10	0.92–1.32
Smoker/smoker	1,756	178	7,008.60	3.57	1.00	
Treatment						
Operation						
Nonsmoker/nonsmoker	17,139	1,136	72,866.98	3.82	0.61	0.52–0.70
Nonsmoker/smoker	599	59	2,682.40	4.17	0.86	0.65–1.15
Smoker/nonsmoker	4,326	256	18,137.76	3.74	0.80	0.67–0.96
Smoker/smoker	3,053	229	12,167.93	3.58	1.00	
Operation with radiotherapy or chemotherapy						
Nonsmoker/nonsmoker	7,159	1,343	27,854.66	3.35	1.09	0.92–1.30
Nonsmoker/smoker	218	48	887.12	3.60	1.31	0.95–1.82
Smoker/nonsmoker	2,252	431	8,125.30	2.91	1.42	1.18–1.71
Smoker/smoker	977	149	3,700.93	3.24	1.00	
Radiotherapy or chemotherapy						
Nonsmoker/nonsmoker	836	224	2,968.45	2.79	0.97	0.68–1.39
Nonsmoker/smoker	28	9	116.82	3.64	0.93	0.45–1.94
Smoker/nonsmoker	346	60	1,167.14	2.78	0.85	0.56–1.29
Smoker/smoker	146	36	517.02	2.91	1.00	

^a^ Adjusted for age at diagnosis, frequency of drinking, Charlson comorbidity index, body mass index, and frequency of physical activity.

## 4 DISCUSSION

In this study, both the prediagnosis and postdiagnosis smoking status were associated with increased mortality among colorectal cancer patients. A cohort study of the CHANCES consortium showed significantly increased mortality among those patients who were former smokers and current smokers before colorectal cancer diagnosis.^13^ A meta‐analysis showed an increased all‐cause mortality among current smokers from 5% to 51% compared to never smokers.^19^ Our results were similar to previous results showing that smoking status before and after colorectal cancer diagnosis was associated with elevated all‐cause mortality.^9^, ^20^, ^21^, ^22^ Additionally, all‐cause mortality among colorectal cancer patients who quit smoking after diagnosis was not significantly different from that in colorectal cancer patients who had continually smoked from the time point before colorectal cancer diagnosis. Although smoking cessation after colorectal cancer diagnosis was not significantly different compared to continual smoking among total population in this study, smoking cessation significantly reduced the mortality among patients who were treated with operation only. Those patients were likely to be in early cancer stage, thus, imply of our study is that smoking cessation after diagnosis may improve survival among patients in early stage colorectal cancer. Our results are inconsistent with a previous population‐based cohort in the United States that reported increased all‐cause mortality among current smokers at the time of breast cancer diagnosis, and continual smokers after diagnosis showed elevated all‐cause mortality, although there was attenuated risk of mortality among quitters after diagnosis.^23^ One possible explanation is that lifetime cumulative exposure to smoking plays a key role in mortality. As our patients’ diagnosis age was more than 60 years, cumulative exposure to smoking before diagnosis was likely to be lifetime exposure and greater than that after diagnosis. Thus, modification of smoking status after colorectal cancer diagnosis may not substantially influence the mortality among smokers at cancer diagnosis. Additionally, these results could be because some patients who quit smoking after diagnosis did so as a result of poor health conditions.^24^

In our results, changes in smoking status after cancer diagnosis were not associated with colorectal cancer‐specific mortality. The association between smoking status and colorectal cancer‐specific mortality may be attenuated since smokers tend to have comorbidities; however, we adjusted for the Charlson comorbidity index.

Smoking cessation after diagnosis was related to a lower risk of mortality than continual smoking among putative early stage cancer patients who underwent surgery only. Previous studies regarding the association between postoperative outcomes and smoking have reported a significantly elevated risk of postoperative mortality among current smokers.^11^, ^25^, ^26^ Similar to these studies, our result showed that those who quit smoking after colorectal cancer diagnosis was associated with a lower risk of mortality than continual smokers. Although the claim data did not contain the stage of colorectal cancer, patients who were treated with only surgery were likely to have early stage disease.^27^, ^28^ Thus, the baseline hazard for mortality would be higher among colorectal cancer patients who underwent surgery with radiotherapy or chemotherapy and among those who were treated with radiotherapy or chemotherapy than among those who received surgery only. Hence, the impact of smoking cessation after diagnosis on the decrease in mortality would not be revealed among colorectal cancer patients who underwent surgery with radiotherapy or chemotherapy due to the high baseline hazard.

Furthermore, smoking before colorectal cancer diagnosis had a greater impact on the increase in mortality than smoking after diagnosis in this study. There have been studies reporting that the treatment effect was not good in patients who were smokers before cancer diagnosis. Thus, a decreased treatment effect due to smoking before diagnosis would worsen the prognosis of colorectal cancer patients.

Our study has several limitations. Since we used claim data, it was not possible to precisely identify all of the factors of the colorectal cancer patients. Thus, we used operational definitions using treatment codes and hospitalization notes with colorectal cancer treatment (Table S1). These assumptions could diminish an overestimation of colorectal cancer incidence. The claim data do not contain information on the stage of colorectal cancer, which is crucial when predicting prognosis. Accordingly, we conducted stratified analyses by treatment. In our study, the survival analysis according to treatment reasonably predicted prognosis (Figure S1). Because we restricted the study population as patients who survived more than 1 year after diagnosis, the study population had a better survival than the total colorectal cancer patients. However, the effect of smoking cessation to prognosis may not be biased because patients who had the worst prognosis could not receive a benefit of smoking cessation on long‐term survival. Despite the limitations above, this study has meaning in that the prediagnosis and postdiagnosis smoking status were considered simultaneously in the analysis to assess the association between smoking and mortality. Previous studies evaluated mortality among colorectal cancer patients in relation to changes in smoking status. However, detailed information on changes in smoking status before and after diagnosis has rarely been considered in previous studies. Furthermore, we conducted stratified analysis according to cancer treatment (operation only, operation with radiotherapy or chemotherapy, and radiotherapy or chemotherapy) to reflect the cancer stage of colorectal cancer patients by regarding cancer treatment as a proxy for cancer stage. A previous study showed that approximately 18% of cancer patients were current smokers, and 10% of those were recent quitters, while 35% of those were former smokers.^29^ In our study, approximately 11% of smokers before colorectal cancer diagnosis continued smoking after colorectal cancer diagnosis, and approximately 19% quit smoking after colorectal cancer diagnosis.

In conclusion, we suggest that the prediagnosis smoking status has a greater impact on all‐cause mortality among colorectal cancer patients. The postdiagnosis smoking status is also associated with elevated mortality among colorectal cancer patients. Additionally, smoking cessation may help to improve survival after diagnosis among colorectal cancer patients who were treated by operation only. Since patients who undergo surgery only are more likely to survive long‐term, smoking cessation is crucial in such patients.

## CONFLICT OF INTEREST

Authors declare that there is no conflict of interest.

## Supporting information

Fig S1

Table S1‐S3

## Data Availability

The data that support the findings of this study are openly available in [The National Health Information Database of the National Health Insurance Service in South Korea] at [https://nhiss.nhis.or.kr], reference number [doi.org/10.1093/ije/dyw253.

## References

[1] Bray F , Ferlay J , Soerjomataram I , Siegel RL , Torre LA , Jemal A . Global cancer statistics 2018: GLOBOCAN estimates of incidence and mortality worldwide for 36 cancers in 185 countries. CA Cancer J Clin. 2018;68:394‐424. 30207593 10.3322/caac.21492

[2] Jung KW , Won YJ , Kong HJ , Lee ES . Cancer statistics in Korea: incidence, mortality, survival, and prevalence in 2016. Cancer Res Treat. 2019;51:417‐430. 30913865 10.4143/crt.2019.138 PMC6473271

[3] Botteri E , Iodice S , Bagnardi V , Raimondi S , Lowenfels AB , Maisonneuve P . Smoking and colorectal cancer: a meta‐analysis. JAMA. 2008;300:2765‐2778. 19088354 10.1001/jama.2008.839

[4] Gong J , Hutter C , Baron JA , et al. A pooled analysis of smoking and colorectal cancer: timing of exposure and interactions with environmental factors. Cancer Epidemiol Biomarkers Prev. 2012;21:1974‐1985. 23001243 10.1158/1055-9965.EPI-12-0692 PMC3493822

[5] Cunningham D , Atkin W , Lenz H‐J , et al. Colorectal cancer. Lancet. 2010;375:1030‐1047. 20304247 10.1016/S0140-6736(10)60353-4

[6] Stanaway JD , Afshin A , Gakidou E , et al. Global, regional, and national comparative risk assessment of 84 behavioural, environmental and occupational, and metabolic risks or clusters of risks for 195 countries and territories, 1990–2017: a systematic analysis for the Global Burden of Disease Study 2017. Lancet. 2018;392:1923‐1994. 30496105 10.1016/S0140-6736(18)32225-6 PMC6227755

[7] The Health Consequences of Smoking‐50 Years of Progress: A Report of the Surgeon General. Atlanta (GA), 2014.

[8] Chao A , Thun MJ , Jacobs EJ , Henley SJ , Rodriguez C , Calle EE . Cigarette smoking and colorectal cancer mortality in the cancer prevention study II. J Natl Cancer Inst. 2000;92:1888‐1896. 11106680 10.1093/jnci/92.23.1888

[9] Tao L , Wang R , Gao YT , Yuan JM . Impact of postdiagnosis smoking on long‐term survival of cancer patients: the Shanghai cohort study. Cancer Epidemiol Biomarkers Prev. 2013;22:2404‐2411. 24319070 10.1158/1055-9965.EPI-13-0805-T PMC3919701

[10] Schnoll RA , Calvin J , Malstrom M , et al. Longitudinal predictors of continued tobacco use among patients diagnosed with cancer. Ann Behav Med. 2003;25:214‐222. 12763716 10.1207/S15324796ABM2503_07

[11] Burke L , Miller LA , Saad A , Abraham J . Smoking behaviors among cancer survivors: an observational clinical study. J Oncol Pract. 2009;5:6‐9. 20856708 10.1200/JOP.0912001 PMC2790635

[12] Mayer DK , Carlson J . Smoking patterns in cancer survivors. Nicotine Tob Res. 2011;13:34‐40. 21097514 10.1093/ntr/ntq199 PMC3001958

[13] Ordóñez‐Mena JM , Walter V , Schöttker B , et al. Impact of prediagnostic smoking and smoking cessation on colorectal cancer prognosis: a meta‐analysis of individual patient data from cohorts within the CHANCES consortium. Ann Oncol. 2018;29:472‐483. 29244072 10.1093/annonc/mdx761 PMC6075220

[14] Cheol Seong S , Kim YY , Khang YH , et al. Data resource profile: the national health information database of the national health insurance service in South Korea. Int J Epidemiol. 2017;46:799‐800. 27794523 10.1093/ije/dyw253 PMC5837262

[15] Lee J , Choe S , Park JW , Jeong SY , Shin A . The risk of colorectal cancer after cholecystectomy or appendectomy: a Population‐based Cohort Study in Korea. J Prev Med Public Health. 2018;51:281‐288. 30514058 10.3961/jpmph.18.105 PMC6283741

[16] Schmoll HJ , Van Cutsem E , Stein A , et al. ESMO Consensus Guidelines for management of patients with colon and rectal cancer. A personalized approach to clinical decision making. Ann Oncol. 2012;23:2479‐2516. 23012255 10.1093/annonc/mds236

[17] Quan H , Sundararajan V , Halfon P , et al. Coding algorithms for defining comorbidities in ICD‐9‐CM and ICD‐10 administrative data. Med Care. 2005;43:1130‐1139. 16224307 10.1097/01.mlr.0000182534.19832.83

[18] Jayasekara H , English DR , Haydon A , et al. Associations of alcohol intake, smoking, physical activity and obesity with survival following colorectal cancer diagnosis by stage, anatomic site and tumor molecular subtype. Int J Cancer. 2018;142:238‐250. 28921583 10.1002/ijc.31049

[19] Walter V , Jansen L , Hoffmeister M , Brenner H . Smoking and survival of colorectal cancer patients: systematic review and meta‐analysis. Ann Oncol. 2014;25:1517‐1525. 24692581 10.1093/annonc/mdu040

[20] Walter V , Jansen L , Hoffmeister M , Ulrich A , Chang‐Claude J , Brenner H . Smoking and survival of colorectal cancer patients: population‐based study from Germany. Int J Cancer. 2015;137:1433‐1445. 25758762 10.1002/ijc.29511

[21] Tamakoshi A , Nakamura K , Ukawa S , et al. Characteristics and prognosis of Japanese colorectal cancer patients: the BioBank Japan Project. J Epidemiol. 2017;27:S36‐S42. 28214186 10.1016/j.je.2016.12.004 PMC5350596

[22] Phipps AI , Baron J , Newcomb PA . Prediagnostic smoking history, alcohol consumption, and colorectal cancer survival: the seattle colon cancer family registry. Cancer. 2011;117:4948‐4957. 21495019 10.1002/cncr.26114 PMC3138819

[23] Parada H , Bradshaw PT , Steck SE , et al. Postdiagnosis changes in cigarette smoking and survival following breast cancer. JNCI Cancer Spectr. 2017;1(1):pkx001. 29608187 10.1093/jncics/pkx001 PMC5875926

[24] Kalkhoran S , Kruse GR , Chang Y , Rigotti NA . Smoking‐Cessation efforts by US adult smokers with medical comorbidities. Am J Med. 2018;131(3):318.e1‐318.e8. 10.1016/j.amjmed.2017.09.025 29024622

[25] Sharma A , Deeb AP , Iannuzzi JC , Rickles AS , Monson JR , Fleming FJ . Tobacco smoking and postoperative outcomes after colorectal surgery. Ann Surg. 2013;258:296‐300. 23059503 10.1097/SLA.0b013e3182708cc5

[26] Jassem J . Tobacco smoking after diagnosis of cancer: clinical aspects. Transl Lung Cancer Res. 2019;8:S50‐S58. 31211105 10.21037/tlcr.2019.04.01 PMC6546630

[27] Society AC . Treatment of colon cancer, by stage. https://www.cancer.org/cancer/colon‐rectal‐cancer/treating/by‐stage‐colon.html. Accessed March 18, 2020.

[28] Society AC . Treatment of rectal cancer, by stage. https://www.cancer.org/cancer/colon‐rectal‐cancer/treating/by‐stage‐rectum.html.

[29] Warren GW , Kasza KA , Reid ME , Cummings KM , Marshall JR . Smoking at diagnosis and survival in cancer patients. Int J Cancer. 2013;132:401‐410. 22539012 10.1002/ijc.27617

