# Supplementary material for: Smoking status before and after colorectal cancer diagnosis and mortality in Korean men: A population‐based cohort study
Source: Cancer Med. 2020 Nov 24;9(24):9641–8. doi: 10.1002/cam4.3609 (PMC7774713; doi:10.1002/cam4.3609)
Supplement: Supplementary file 1 — Fig S1 [file CAM4-9-9641-s001.pdf]

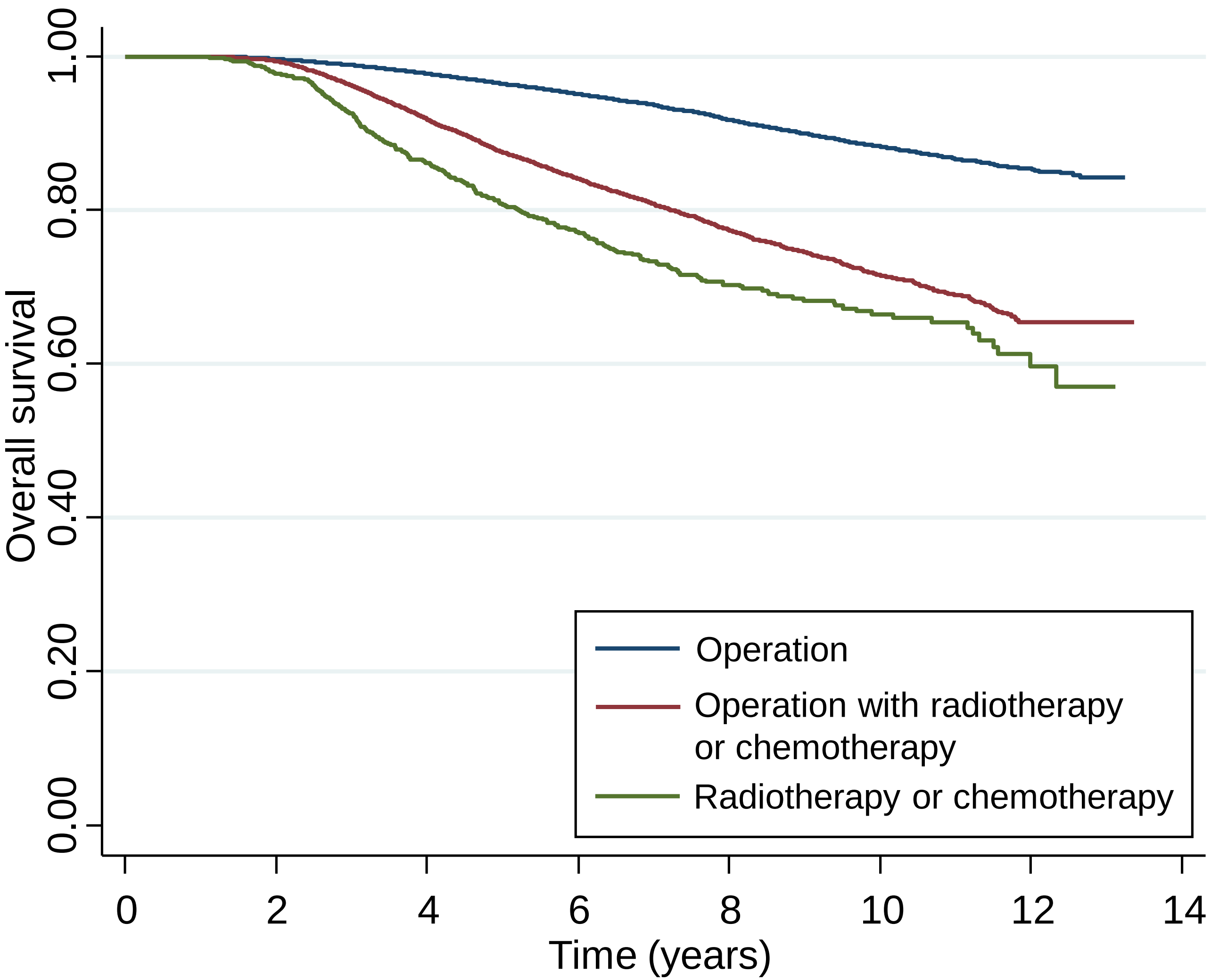


Number at risk

| | | | | | | | | |
|---|---|---|---|---|---|---|---|---|
| Operation | 25117 | 24929 | 20584 | 13997 | 8015 | 3883 | 866 | 0 |
| Operation with radiotherapy or chemotherapy | 10606 | 10432 | 8119 | 5332 | 3122 | 1423 | 211 | 0 |
| Radiotherapy or chemotherapy | 1356 | 1309 | 955 | 619 | 328 | 157 | 37 | 0 |
