# Supplementary material for: Smoking status before and after colorectal cancer diagnosis and mortality in Korean men: A population‐based cohort study
Source: Cancer Med. 2020 Nov 24;9(24):9641–8. doi: 10.1002/cam4.3609 (PMC7774713; doi:10.1002/cam4.3609)
Supplement: Supplementary file 2 — Table S1‐S3 [file CAM4-9-9641-s002.docx]

| Supplementary table 1. List of claim codes for colorectal cancer treatments in the National Health Insurance Service | |
| --- | --- |
| **Treatment** | **Claim codes** |
| **Operation** |  |
| Rt. or Lt. hemicolectomy | QA671, Q2671 |
| Subtotal colectomy | Q1261, Q1262 |
| Total colectomy | QA672, Q2672 |
| Segmental resection | QA673, Q2673 |
| Colectomy with proximal colostomy and distal stump | QA679, Q2679 |
| Transanal rectal tumor resection | Q2891 |
| Transsacral or parasacral rectal tumor resection | Q2890 |
| Abdominal approach rectal tumor resection | Q2892 |
| Transanal endoscopic microsurgery of rectal tumor resection | Q2893 |
| Anterior resection | QA921, Q2921 |
| Low anterior Resection | Q2927, QA922, Q2922 |
| Abdominoperineal resection | QA923, A2923 |
| Abdominal pull-through operation | QA924, A2924 |
| Total coloprotectomy with ileostomy | QA925, A2925 |
| Total coloprotectomy with ileal pouch-anal anastomosis | QA926, A2926 |
| **Chemotherapy** |  |
| Capecitabine | 122701ATB, 122702ATB |
| 5-FU | 161430BIJ, 161431BIJ, 161432BIJ |
| Leucovorin | 566132BIJ, 566134BIJ, 622630BIJ, 622631BIJ, 622632BIJ, 521001BIJ, 521002BIJ |
| Irinotecan | 177430BIJ, 177431BIJ, 177432BIJ, 177433BIJ, 177434BIJ, 177435BIJ, 177436BIJ, 177437BIJ |
| Bevacizumab | 554330BIJ, 554331BIJ |
| Cetuximab | 556430BIJ |
| Oxaliplatin | 205830BIJ, 205834BIJ, 205803BIJ, 205832BIJ |
| **Radiotherapy** |  |
|  | HD051, HD054, HD052, HD055, HD053, HD056, HD057, HD058, HD059, HD061, HD071, HD072, HD073, HD080, HD081, HD082, HD083, HD084, HD085, HD086, HD087, HD088, HD089 |

| Supplementary table 2. Association between pre- and post-diagnosis status of smoking and all-cause mortality among subjects who have health examination within 1 year before colorectal cancer diagnosis | | | | | | |
| --- | --- | --- | --- | --- | --- | --- |
| Smoking status | No. of patients (N=28,863) | No. of deaths (N=2,930) | Person-years | Follow-up (median) | HR^†^ | 95% CI |
| **Prediagnosis** |  |  |  |  |  |  |
| Nonsmoker | 20,161 | 2,052 | 133,253.76 | 6.27 | 1.00 |  |
| Smoker | 8,702 | 878 | 56,374.58 | 6.12 | 1.23 | 1.13-1.34 |
| Duration of smoking, years^‡^ |  |  |  |  |  |  |
| <10 | 4,215 | 352 | 22,274.94 | 5.25 | 1.24 | 1.10-1.39 |
| 10-19 | 945 | 66 | 6,552.79 | 6.60 | 1.46 | 1.14-1.88 |
| 20-29 | 2,042 | 158 | 13,360.58 | 6.21 | 1.42 | 1.20-1.68 |
| ≥30 | 1,500 | 302 | 14,186.26 | 9.65 | 1.12 | 0.99-1.27 |
| **Postdiagnosis** |  |  |  |  |  |  |
| Nonsmoker | 24,921 | 2,530 | 102,163.69 | 3.64 | 1.00 |  |
| Smoker | 3,942 | 400 | 15,748.33 | 3.59 | 1.17 | 1.05-1.31 |
| **Prediagnosis/postdiagnosis** |  |  |  |  |  |  |
| Nonsmoker/nonsmoker | 19,530 | 1,972 | 80,780.29 | 3.68 | 1.00 |  |
| Nonsmoker/smoker | 631 | 80 | 2,748.99 | 4.00 | 1.25 | 1.00-1.57 |
| Smoker/nonsmoker | 5,391 | 558 | 21,383.40 | 3.54 | 1.29 | 1.17-1.42 |
| Duration of smoking, years^‡^ |  |  |  |  |  |  |
| <10 | 2,528 | 224 | 7,624.73 | 2.67 | 1.24 | 1.08-1.43 |
| 10-19 | 642 | 47 | 2,933.59 | 3.78 | 1.48 | 1.10-1.99 |
| 20-29 | 1,342 | 119 | 5,485.16 | 3.62 | 1.57 | 1.30-1.91 |
| ≥30 | 879 | 168 | 5,339.93 | 6.57 | 1.19 | 1.01-1.39 |
| Smoker/smoker | 3,311 | 320 | 12,999.34 | 3.55 | 1.25 | 1.10-1.41 |
| Duration of smoking, years^‡^ |  |  |  |  |  |  |
| <10 | 1,687 | 128 | 5,044.90 | 2.76 | 1.18 | 0.98-1.41 |
| 10-19 | 303 | 19 | 1,280.61 | 3.70 | 1.52 | 0.97-2.40 |
| 20-29 | 700 | 39 | 2,766.04 | 3.55 | 1.19 | 0.86-1.64 |
| ≥30 | 621 | 134 | 3,907.79 | 6.71 | 1.31 | 1.10-1.57 |
| ^†^Adjusted for age at diagnosis, frequency of drinking, Charlson comorbidity index, body mass index, frequency of physical activity, and treatment.  ^‡^Prediagnosis duration of smoking. | | | | | | |

| Supplementary table 3. Association between pre- and post-diagnosis status of smoking and colorectal cancer mortality among male colorectal cancer patients | | | | | | |
| --- | --- | --- | --- | --- | --- | --- |
| Smoking status | No. of patients (N=37,079) | No. of colorectal cancer deaths (N=2,137) | Person-years | Follow-up (median) | HR^†^ | 95% CI |
| **Prediagnosis** |  |  |  |  |  |  |
| Nonsmoker | 25,979 | 1,510 | 173,026.02 | 6.36 | 1.00 |  |
| Smoker | 11,100 | 627 | 72,516.93 | 6.20 | 1.08 | 0.98-1.19 |
| Duration of smoking, years^‡^ |  |  |  |  |  |  |
| <10 | 5,180 | 240 | 27,081.25 | 5.16 | 1.06 | 0.92-1.22 |
| 10-19 | 1,209 | 61 | 8,552.68 | 6.95 | 1.32 | 1.02-1.72 |
| 20-29 | 2,536 | 143 | 16,833.66 | 6.33 | 1.33 | 1.11-1.60 |
| ≥30 | 2,175 | 183 | 20,049.34 | 9.46 | 0.93 | 0.79-1.08 |
| **Postdiagnosis** |  |  |  |  |  |  |
| Nonsmoker | 32,058 | 1,919 | 131120.29 | 3.63 | 1.00 |  |
| Smoker | 5,021 | 218 | 20072.28 | 3.58 | 0.84 | 0.73-0.97 |
| **Prediagnosis/postdiagnosis** |  |  |  |  |  |  |
| Nonsmoker/nonsmoker | 25,134 | 1,468 | 103690.09 | 3.66 | 1.00 |  |
| Nonsmoker/smoker | 845 | 42 | 3686.39 | 3.99 | 0.86 | 0.63-1.17 |
| Smoker/nonsmoker | 6,924 | 451 | 27430.20 | 3.54 | 1.21 | 1.08-1.35 |
| Duration of smoking, years^‡^ |  |  |  |  |  |  |
| <10 | 3,133 | 165 | 9233.50 | 2.62 | 1.12 | 0.95-1.32 |
| 10-19 | 813 | 47 | 3733.26 | 4.00 | 1.38 | 1.02-1.86 |
| 20-29 | 1,671 | 113 | 6836.01 | 3.64 | 1.49 | 1.22-1.83 |
| ≥30 | 1,307 | 126 | 7627.43 | 6.23 | 1.11 | 0.93-1.34 |
| Smoker/smoker | 4,176 | 176 | 16385.89 | 3.55 | 0.89 | 0.76-1.04 |
| Duration of smoking, years^‡^ |  |  |  |  |  |  |
| <10 | 2,047 | 75 | 5965.80 | 2.70 | 0.95 | 0.75-1.20 |
| 10-19 | 396 | 14 | 1656.83 | 3.70 | 1.23 | 0.72-2.08 |
| 20-29 | 865 | 30 | 3466.05 | 3.59 | 0.96 | 0.66-1.38 |
| ≥30 | 868 | 57 | 5297.21 | 6.58 | 0.76 | 0.58-0.99 |
| ^†^Adjusted for age at diagnosis, frequency of drinking, Charlson comorbidity index, body mass index, frequency of physical activity, and treatment.  ^‡^Prediagnosis duration of smoking. | | | | | | |
